# Disseminated Cutaneous Tophaceous Gout in a Man With Advanced HIV Disease

**DOI:** 10.7759/cureus.117979

**Published:** 2026-10-15

**Authors:** Ruan S de Jager, Caitlin Iyer, Susanna M Kannenberg

**Affiliations:** 1 Dermatology, Stellenbosch University, Cape Town, ZAF; 2 Dermatology, University of Cape Town, Cape Town, ZAF

**Keywords:** advanced hiv disease, allopurinol, cutaneous gout, dermatopathology, diagnostic delay, hiv, hyperuricemia, monosodium urate, tophaceous gout

## Abstract

Cutaneous tophi typically develop at periarticular sites in patients with longstanding gout. Widespread dermal deposition of monosodium urate beyond these sites is uncommon and is not recognized as a manifestation of human immunodeficiency virus (HIV) infection. We report a 54-year-old man with advanced HIV disease who presented with a ten-year history of progressively enlarging nodules on both lower limbs. HIV had been diagnosed in 2019, and he had been virologically suppressed on tenofovir disoproxil fumarate, lamivudine, and dolutegravir since 2020, although his CD4 count remained 34 cells/µL. The lesions were asymptomatic and had not previously been investigated. Examination showed numerous firm, hyperpigmented papules and nodules across both lower limbs together with bilateral olecranon tophi. Serum urate was 0.61 mmol/L, renal function was mildly reduced, and body mass index was 41 kg/m². Punch biopsy demonstrated dermal deposits of amorphous, feathery eosinophilic material bordered by a granulomatous infiltrate, and stains for mycobacteria and fungi were negative. No tissue cultures were obtained; disseminated cutaneous tophaceous gout was diagnosed by clinicopathologic correlation rather than crystal identification. Allopurinol was restarted with dose escalation and colchicine prophylaxis. At six months, the serum urate had fallen to 0.40 mmol/L, within the laboratory reference range but above the target for tophaceous gout, and the tophi had not regressed. This case shows how asymptomatic skin disease can go uninvestigated for years in a patient with a demanding chronic illness. We report the two conditions as coexisting and do not suggest that HIV caused the cutaneous tophi.

## Introduction

Gout is the most common inflammatory arthritis, and sustained hyperuricemia leads to deposition of monosodium urate crystals in and around joints. Tophi, the macroscopic aggregates of these crystals, characteristically form at periarticular sites, the helix of the ear, and the olecranon bursa. Deposition within the dermis at sites remote from joints is uncommon, and the resulting papulonodular eruption closely resembles infectious, granulomatous, and neoplastic dermatoses [1,2]. Published cases of disseminated cutaneous gout describe patients with longstanding, poorly controlled disease, and reported predisposing factors include chronic renal impairment, exogenous glucocorticoids, and post-transplant immunosuppression [2-5].

The difficulty is greater in an immunocompromised patient. A chronic papulonodular eruption in advanced human immunodeficiency virus (HIV) disease will reasonably prompt consideration of cutaneous tuberculosis, deep fungal infection, and cutaneous dissemination of systemic mycoses, particularly where those diseases are common, while noninfectious conditions such as acquired perforating dermatosis share both the morphology and the metabolic risk profile. Biopsy is usually needed to separate them. Hyperuricemia is common in people living with HIV, and gout in this population occurs against a background of obesity, hypertension, and renal impairment [6-8]. Disseminated cutaneous tophi, however, are not a recognized manifestation of HIV infection. We searched PubMed/Medical Literature Analysis and Retrieval System Online (MEDLINE) and Google Scholar from inception to September 2026, combining the terms “cutaneous gout,” “disseminated cutaneous gout,” “cutaneous tophi,” “tophaceous gout,” and “miliarial gout” with “HIV” and “AIDS,” and hand-searched the reference lists of the reports retrieved. That search found no previously reported case of disseminated cutaneous tophaceous gout in a patient with advanced HIV disease. The claim reflects our own search and not a systematic review. We describe a patient with advanced HIV disease in whom widespread cutaneous tophi accumulated over a decade without being investigated. The two conditions coexisted in this man, and we infer no causal link between them.

## Case presentation

A 54-year-old man was referred to the dermatology service with a ten-year history of progressively enlarging nodules on both lower limbs. He had received a diagnosis of HIV in 2019 at an advanced stage, and antiretroviral therapy with tenofovir disoproxil fumarate, lamivudine, and dolutegravir was commenced in 2020. Adherence was good, and the viral load was suppressed below 20 copies/mL, but the CD4 count remained 34 cells/µL, indicating immunologic non-response.

The lesions were asymptomatic throughout. He did not raise them at his antiretroviral clinic visits, no biopsy had been performed, and no diagnosis had been reached before referral. Some years earlier he had experienced vague, poorly localized joint pain rather than a classical acute monoarthritis, and an olecranon tophus was noted incidentally during that assessment. Gout was diagnosed clinically; no joint aspiration was performed, and crystals were never identified. Allopurinol was prescribed at the time, but his account of what followed was inconsistent, and he appears to have taken it only intermittently. He reported no further episodes of joint pain. One serum urate measurement of 0.5 mmol/L survives from that period. At dermatologic referral, the working differential included cutaneous tuberculosis, deep fungal infection, and acquired perforating dermatosis.

On examination, there were numerous firm, hyperpigmented papules and nodules across both lower limbs, ranging from 5 mm to 20 mm, several with central crusting and erosion (Figures 1, 2). Firm, nontender subcutaneous nodules were present at both olecranon regions. The digits, first metatarsophalangeal joints, ear helices, trunk, and mucosae were spared. Body mass index was 41 kg/m², and blood pressure was 140/84 mmHg. He took no diuretics, drank alcohol only socially, and had no family history of gout.

**Figure 1:**
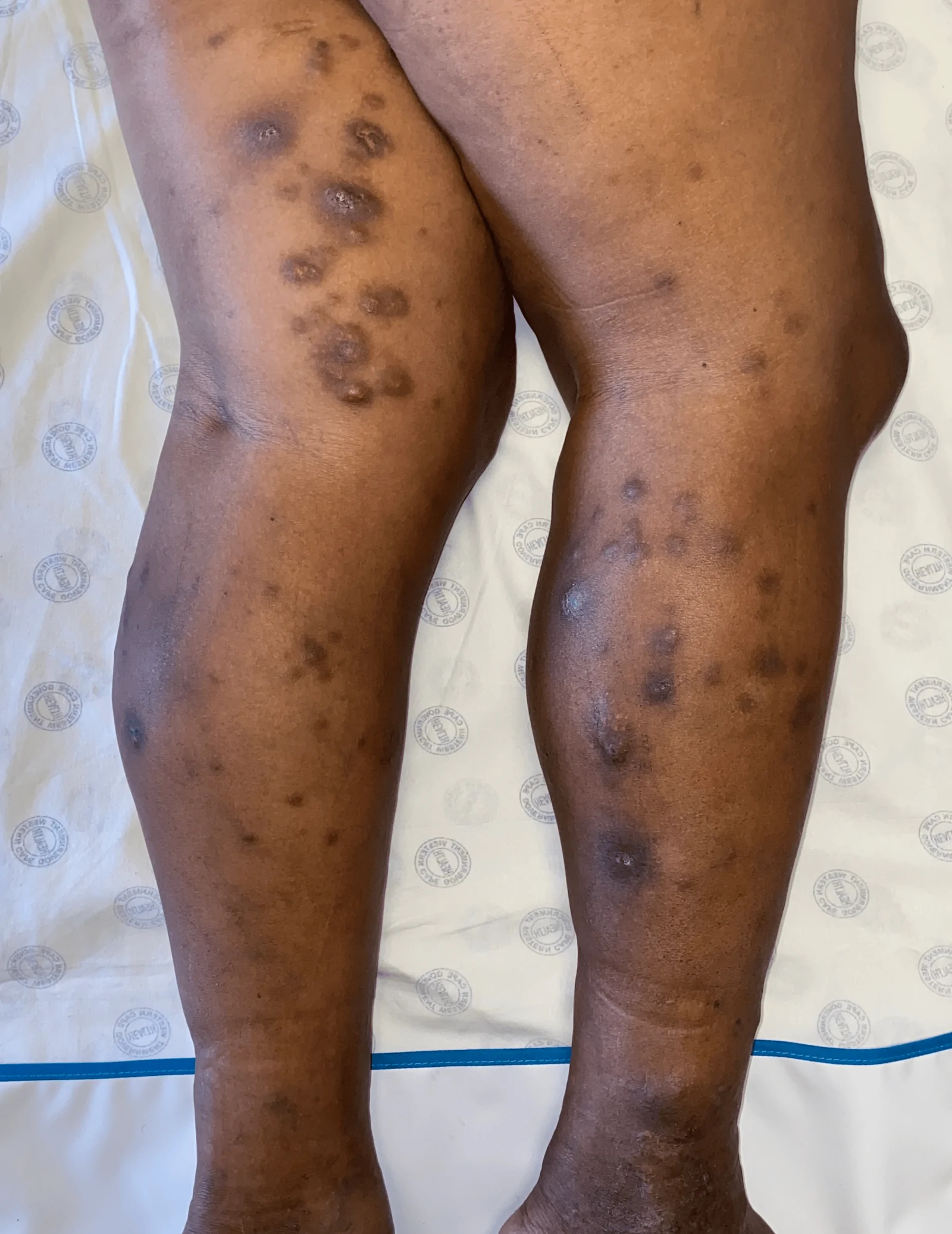
Distribution of cutaneous tophi. Both lower limbs show multiple firm, hyperpigmented papules and nodules of varying size scattered across the calves.

**Figure 2:**
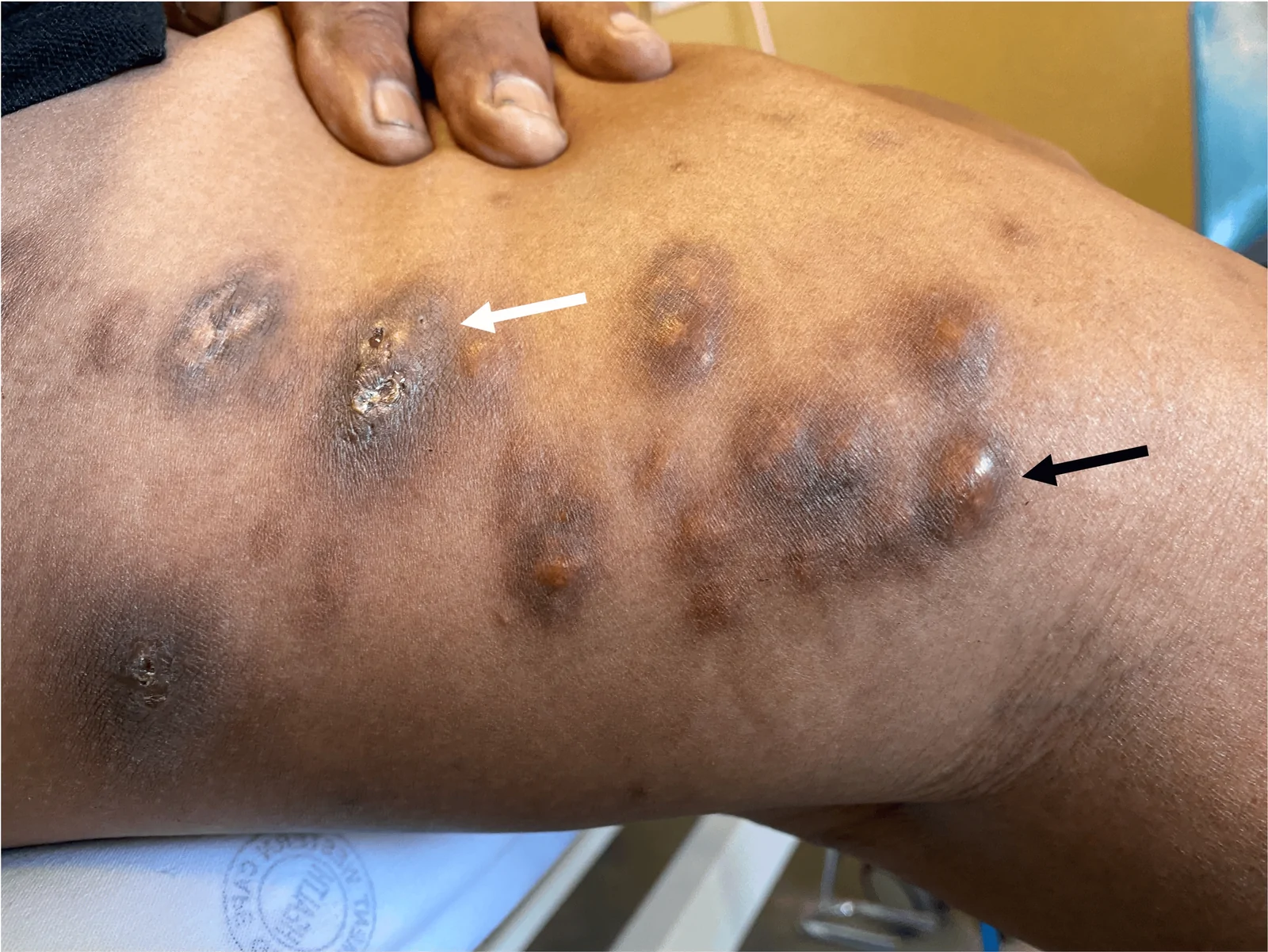
Lesion morphology. Close-up of a representative affected area showing firm, dome-shaped nodules of approximately 5 to 20 mm, several grouped and partly coalescing into plaques. Arrows indicate an intact, glossy nodule (black) and a lesion with central crusting and erosion (white).

Serum urate was elevated at 0.61 mmol/L (reference range 0.21-0.43 mmol/L). Creatinine was 109 µmol/L (reference range 64-104 µmol/L), giving an estimated glomerular filtration rate of 66 mL/min/1.73 m². Glycated hemoglobin was 6.2%. The white cell count was 13.49 × 10⁹/L (reference range 3.92-10.40 × 10⁹/L) with neutrophilia, and C-reactive protein was 6 mg/L. Serum calcium, alanine transaminase, and electrolytes were within reference ranges.

Punch biopsy of a lower limb nodule showed dermal deposits of amorphous, feathery eosinophilic material bordered by a granulomatous infiltrate containing palisading histiocytes and multinucleated foreign-body giant cells (Figure 3). Ziehl-Neelsen, periodic acid-Schiff, and Grocott methenamine silver stains were negative. No tissue was sent for mycobacterial or fungal culture, and nucleic acid amplification testing was not performed. Polarized microscopy of aspirated or expressed material was not performed, as it is not routinely available at our institution. Taken with the hyperuricemia, the concurrent olecranon tophi, and the clinical morphology, these findings supported a diagnosis of disseminated cutaneous tophaceous gout, established by clinicopathologic correlation rather than by crystal identification.

**Figure 3:**
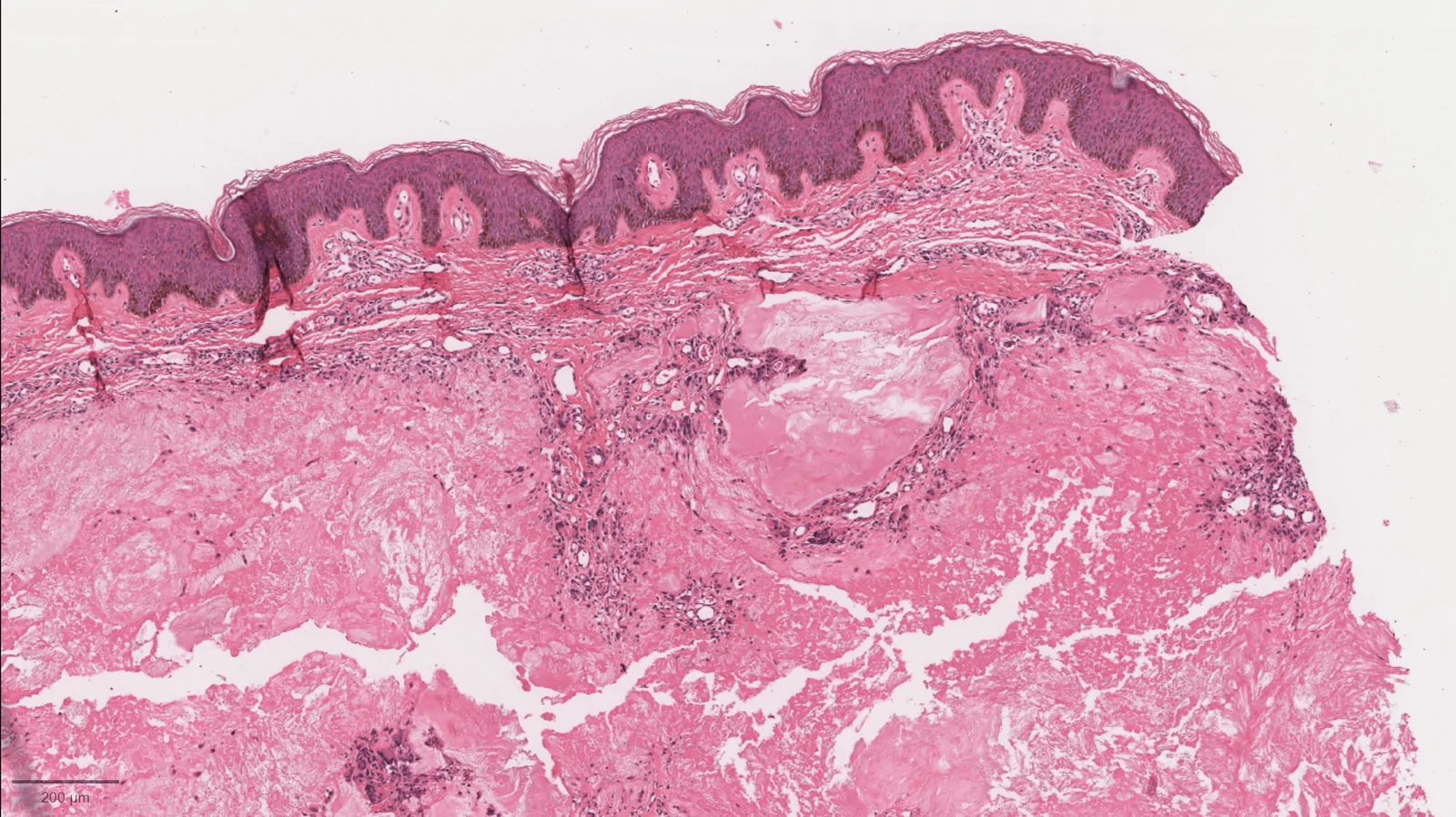
Histopathology of a cutaneous tophus. Punch biopsy of a lower limb nodule showing dermal deposits of amorphous, feathery eosinophilic material bordered by a granulomatous infiltrate (hematoxylin and eosin, ×40).

Allopurinol was restarted at 100 mg daily, with colchicine 0.5 mg twice daily for flare prophylaxis, intended to continue for at least six months and for as long as flares occurred during titration. At the two-month review, the serum urate had barely moved, from 0.61 to 0.6 mmol/L, and allopurinol was increased to 200 mg daily. He missed the next appointment but continued the medication. When seen at six months, the urate had fallen to 0.4 mmol/L, still above the 0.36 mmol/L target recommended for tophaceous gout; the dose should have been raised again at that visit and was not. Neither the cutaneous nor the olecranon tophi had changed, although this was a qualitative assessment, with no lesion counts, dimensions, or serial photographs recorded. No treatment-related adverse effects were reported. He did not attend thereafter and was lost to follow-up.

## Discussion

The ten-year delay is not hard to explain. The nodules were painless. The patient did not mention them at his antiretroviral visits, and the focus at those visits was properly on virologic control and immune reconstitution. No acute attack of gout ever forced the question, and the olecranon tophus noted years earlier went unexamined once the allopurinol lapsed. A nodular eruption that neither hurts nor is mentioned is easily deferred at each successive visit, and deposition continued throughout.

A chronic papulonodular eruption on the limbs of a man with a CD4 count of 34 cells/µL raises infection first, and reasonably so. Cutaneous tuberculosis is common in our setting, and in advanced immunosuppression it may present as a gumma or as disseminated disease rather than in its classical forms. Deep fungal infection belongs in the same differential, and acquired perforating dermatosis was considered because several lesions were crusted and eroded and the patient had both prediabetes and mildly reduced renal function. What argued against all three, before any stain was read, was the tempo. Ten years is slow. The eruption caused him nothing, there were no constitutional symptoms, and the C-reactive protein was 6 mg/L. Nodules at both olecranon regions and a serum urate of 0.61 mmol/L pointed elsewhere. Table 1 sets out the findings that favored or excluded each diagnosis.

**Table 1:** Differential diagnosis of a chronic papulonodular eruption in a patient with advanced HIV disease, and the findings that favored or excluded each diagnosis in this patient.

Diagnosis	Clinical findings in this patient	Histopathologic findings in this patient
Disseminated cutaneous tophaceous gout (final diagnosis)	Supporting: ten-year progressive, asymptomatic course; bilateral firm nodules 5–20 mm; concurrent olecranon tophi; serum urate 0.61 mmol/L; obesity, borderline hypertension, mild renal impairment; olecranon tophus documented years earlier	Supporting: dermal deposits of amorphous, feathery eosinophilic material bordered by palisading histiocytes and multinucleated foreign-body giant cells
Cutaneous tuberculosis	Against: decade-long asymptomatic course; no constitutional symptoms; C-reactive protein 6 mg/L; no sinus tracts, undermined ulceration, or lymphadenopathy	Against: foreign-body rather than caseating granuloma; no Langhans giant cells or caseation necrosis; Ziehl-Neelsen negative, though of limited sensitivity in advanced HIV
Deep fungal infection	Against: indolent decade-long course; bilateral symmetry; no lymphocutaneous chain, verrucous plaque, or umbilicated papules; no systemic illness	Against: no fungal elements, yeast forms, or sclerotic bodies on periodic acid-Schiff or Grocott methenamine silver staining; no suppurative granulomatous inflammation
Acquired perforating dermatosis	For: prediabetes, mildly reduced renal function, central crusting and erosion. Against: lesions painless rather than pruritic; up to 20 mm and firmly nodular; neither established diabetes nor advanced chronic kidney disease	Against: no cup-shaped epidermal invagination or keratotic plug; dermal material amorphous and feathery rather than degenerate collagen or elastic fiber in transepidermal elimination

The biopsy settled it. The amorphous eosinophilic deposits represent monosodium urate dissolved during routine formalin fixation, which leaves the characteristic feathery clefts [1,4]. The surrounding reaction was of foreign-body type, with palisading histiocytes and giant cells arranged around deposited material, not the caseating granuloma of cutaneous tuberculosis or the neutrophilic microabscesses of deep fungal infection, and there was no keratotic plug or transepidermal elimination to suggest a perforating dermatosis.

These findings are not of equal value. The positive one carries the diagnosis. The negative stains carry less than they appear to, because granulomas are often poorly formed at this degree of immunosuppression, and Ziehl-Neelsen is insensitive in that setting. Culture or nucleic acid amplification would have been more secure, and neither was sent. The clinical course offers some reassurance where the laboratory cannot, in that ten years of untreated disseminated mycobacterial or fungal infection would not have left this man systemically well. Even so, the diagnosis rests on clinicopathologic correlation and not on crystal identification. Polarized microscopy of aspirated or expressed material remains the reference standard. Demonstrating urate in tissue requires fixation in absolute alcohol or a de Galantha stain, and since this specimen went into formalin, that option has closed.

Disseminated cutaneous gout is itself uncommon. A review of 23 reported cases found most patients to be middle-aged men of Asian or Hispanic ancestry with longstanding, poorly controlled gout and moderate to severe renal impairment [2]. Individual reports describe tophi mistaken for xanthomas and sebaceous tumors in iatrogenic Cushing syndrome [3], ulcerated deposits in renal impairment [4], plantar tophi resembling viral warts after transplantation [5], and skin-colored plaques with punched-out depressions on the trunk and limbs [1]. Our patient fits none of this closely. He had no documented attack of acute gouty arthritis, only mild renal impairment, and no exposure to diuretics or glucocorticoids. His cutaneous burden was also out of proportion to modest olecranon disease, the reverse of the usual pattern, and that disproportion is part of why gout was not considered.

Whether HIV contributed to urate deposition here cannot be settled by one case. Hyperuricemia is common in this population: a cohort predating combination antiretroviral therapy found it in 41% of HIV-positive individuals and in none of the HIV-negative controls with similar risk factors [6]. Increased purine turnover during active viremia and reduced renal urate clearance have both been proposed, and urate elevation has been linked to particular antiretroviral agents [7]. A more recent case-control study placed the emphasis differently, finding conventional risk factors, hypertension above all, driving gout in an HIV cohort rather than anything HIV-specific [8]. That is closer to our patient. Severe obesity, borderline hypertension, prediabetes, and mildly reduced renal function are, between them, sufficient to account for sustained hyperuricemia. His infection was advanced when diagnosed in 2019 and had therefore gone untreated for some preceding period we cannot quantify, but any contribution from the virus, from its treatment, or from the associated renal impairment is speculative. We report the two conditions as coexisting and draw no causal inference.

A serum urate of 0.4 mmol/L sits inside most laboratory reference ranges and looks, on a results screen, like a treated patient. It is above the 0.36 mmol/L target recommended for tophaceous gout, and established tophi need sustained sub-target urate for one to two years before they visibly shrink [9]. Our own practice shows how easily the reference range is mistaken for the target: allopurinol reached 200 mg daily, well short of the doses commonly needed to reach the target, and went no higher.

This patient’s intermittent use of allopurinol through a symptom-free interval is a familiar obstacle, since nothing reinforces a diagnosis that causes no pain. His antiretroviral therapy tells a different story. He took it reliably, and his viral load shows it. The condition with a clinic, a schedule, and a number to watch was the one that was treated.

Images of dermatologic disease in darker skin phototypes remain underrepresented in the published literature [10], and the photographs here document this presentation in Fitzpatrick skin type IV [11].

This report has real limitations. Crystals were never identified, so the diagnosis is inferential. Infection was addressed by special stains alone, which is not the same as excluding it. The history before referral is retrospective and partly unverifiable: the earlier diagnosis of gout was clinical, the duration of allopurinol therapy cannot be stated, and only one historical urate measurement survives. The assessment at six months was qualitative, without measurement or serial photography. Urate-lowering therapy was under-titrated and never reached target. He did not return after six months, so the long-term cutaneous response is unknown. A single case cannot show whether HIV contributed to the development of cutaneous tophi, and we do not suggest that it did.

## Conclusions

A chronic nodular eruption in a profoundly immunocompromised patient invites an infective explanation, and where those infections are common, that instinct is usually right. It should not be the only one. Noninfectious causes, metabolic and depositional among them, deserve the same consideration, particularly when the course runs over years, the lesions are painless, and the patient is otherwise well. A serum urate is a cheap test, and in this man it was abnormal.

The diagnosis here was reached by clinicopathologic correlation and not by crystal identification. No cultures were sent; negative stains do not exclude infection at a CD4 count of 34 cells/µL, and the formalin-fixed specimen can no longer supply the crystals. We regard the diagnosis as secure on the combination of characteristic histopathology, hyperuricemia, and concurrent olecranon tophi, while accepting that it falls short of the reference standard.

We report advanced HIV disease and disseminated cutaneous tophaceous gout as coexisting in one patient, and offer the case as a diagnostic consideration rather than as evidence that either condition caused the other. Painless skin disease in a patient with a demanding chronic illness can go unexamined for years. And once gout is confirmed, urate-lowering therapy should be titrated to the target set for tophaceous disease rather than to the laboratory reference range, a target our own patient never reached.
